# On the Track of a Ward-Maid

**Published:** 1920-04-03

**Authors:** 


### ON THE TRACK OF A WARD-MAID.

[We have pleasure in publishing these engaging verses, but beg to acknowledge that we have altered the title, removed one split infinitive, and wish to apologise to the author for omitting four stanzas.—Ed. The Hospital.]

You start with a simple equipment,
A pencil, a little black book,
An air of command and assurance,
And, of course, your most dignified look.

You've got a few names and addresses,
Your plans are all carefully laid,
You've only to pick where you fancy.
Quite simple, this choosing a maid.

You make for the first one, whose record
Sounds almost too good to be true,
And, after a quiet inspection,
Decide that, perhaps, she will do.

But she's one of our unemployed workers,
And benefits now are not small.
Besides, "What's a common domestic?"
No. That would not suit her at all.

In a second attempt you endeavour
A diplomat's tactics to try—
"You know of a splendid appointment
And really think she should apply."

No use. So you try yet another,
And track them down trail after trail,
Suggesting, cajoling, imploring—
But nothing's of any avail.

You wildly rush hither and thither,
To districts unheard of before;
And it's certain to be the last building,
And always the very top floor.

You stumble up creaky old stairways,
And breathlessly beg to be heard,
But read in their faces—refusal—
Before you have uttered a word.

Just a maid! And you pass them in hundreds,
But can't lay your fingers on one;
For you daren't fish her out with a boat-hook,
Or marshal her in with a gun.

If you're lucky, you may persuade someone
To come to the rescue at last,
But don't for a moment imagine
The glory of conquest is past.

You must pet her and pamper her fondly,
Anticipate ev'ry request,
Make sure that she gets her "off duty,"
And rations are fullest and best.

She likes to be just ornamental,
And only to work when inclined,
And you, with the tact of an Angel,
Pretend that you really don't mind.

She tells you her wishes, regarding
Her salary, duties, and dress;
Your part is quietly to listen,
And then, with a smile, acquiesce.

And never, by look, word or action,
Your wrath or displeasure make known,
Or you're certain on rising next morning
To find that your Treasure has flown.

Sister Ishbel.

